# GPs' opinions of public and industrial information regarding drugs: a cross-sectional study

**DOI:** 10.1186/1472-6963-11-204

**Published:** 2011-08-25

**Authors:** Ingmarie Skoglund, Cecilia Björkelund, Kirsten Mehlig, Ronny Gunnarsson, Margareta Möller

**Affiliations:** 1 Primary Care Unit, Department of Public Health and Community Medicine, University of Gothenburg, Gothenburg, Sweden; 2 Research and Development Unit, Southern Älvsborg County, Borås, Sweden; 3 Public Health Epidemiology Unit, Department of Public Health and Community Medicine, University of Gothenburg, Gothenburg, Sweden; 4 Centre for Health Care Sciences, Örebro County Council, Örebro University, Örebro, Sweden

## Abstract

**Background:**

General Practitioners {GP} in Sweden prescribe more than 50% of all prescriptions. Scientific knowledge on the opinions of GPs regarding drug information has been sparse. Such knowledge could be valuable when designing evidence-based drug information to GPs. GPs' opinions on public- and industry-provided drug information are presented in this article.

**Methods:**

A cross-sectional study using a questionnaire was answered by 368 GPs at 97 primary-health care centres {PHCC}. The centres were invited to participate by eight out of 29 drug and therapeutic committees {DTCs}. A multilevel model was used to analyse associations between opinions of GPs regarding drug information and whether the GPs worked in public sector or in a private enterprise, their age, sex, and work experience. PHCC and geographical area were included as random effects.

**Results:**

About 85% of the GPs perceived they received too much information from the industry, that the quality of public information was high and useful, and that the main task of public authorities was to increase the GPs' knowledge of drugs. Female GPs valued information from public authorities to a much greater extent than male GPs. Out of the GPs, 93% considered the main task of the industry was to promote sales. Differences between the GPs' opinions between PHCCs were generally more visible than differences between areas.

**Conclusions:**

Some kind of incentives could be considered for PHCCs that actively reduce drug promotion from the industry. That female GPs valued information from public authorities to a much greater extent than male GPs should be taken into consideration when designing evidence-based drug information from public authorities to make implementation easier.

## Background

In Sweden, General Practitioners {GPs} comprise the largest group of prescribers, writing more than 50% of all prescriptions [1]. As costs for medication have risen, there has been an increasing need for finding ways to get better value for money [2,3]. Focus has increased on evidence-based medicine that refers to the conscientious, distinct and sensible use of the most reliable and current knowledge when making decisions affecting individual patients [4,5]. There has been much debate on how the pharmaceutical industry influences physicians and assessment agencies [6-9]. Physicians' attitudes continue to be positive towards industry-related activities according to an American hospital study [9]. Published studies with companies as sponsors are more likely to present results that favour the company [8] and it has been claimed that the financial arrangements with industry are well hidden [7]. The pharmaceutical industry's financial contribution to continuing education {CME} of Swedish physicians has been estimated at one billion Swedish crowns (€104.6 million) [10]. Swedish employers, mainly in the public sector are said to contribute 67% of the total cost of CME [11]. However, these figures are based on weak ground due to trade secrecy, but the best available in literature.

The development of drug and therapeutic committees {DTCS} has varied considerably in Europe and has been particularly extensive in the Nordic countries [12,13]. In Sweden they began in the 1960s in hospital settings as collaboration between clinicians, pharmacists and clinical pharmacologists. Focus was broadened in the 1980s to increase the commitment of GPs.

Since 1996 a Swedish law states that each county council is obliged to have at least one DTC whose overall aim is to promote the rational use of drugs based on evidence-based principles of drug therapy on all levels of the health care system. This is achieved through the selection of recommended drugs and support of using them through education and information in academic drug detailing, often provided by pharmacists or physicians. The DTCs have worked within multidisciplinary networks including GPs and other specialized physicians, district and other specialized nurses, and pharmacists.

Attitudes on new drugs were explored among high and low prescribing GPs in the UK. Acquired knowledge and subjective and ideological beliefs were regarded as influential on prescribing among British GPs [14]. Hospital physicians, pharmaceutical representatives, prescribing advisers and cost considerations were in varying degrees influential on British GPs. There were notable differences between high and low prescribers of new pharmaceuticals. The impact of cost on prescribing behaviour did not represent a significant barrier to uptake of new medicines and seemed to be of lower importance than safety and efficacy. Limiting the role of the companies could be necessary to enable cost control [15]. Results from studies on GPs throughout the world suggest that it is necessary to limit the role of pharmaceutical companies in physician training and to emphasize more objective sources of information [16-21]. The personal information on drugs might be more important than the message as was proposed in an Australian qualitative study [22]. Knowledge is sparse of GPs' opinions and attitudes regarding drug information services in general, and, in particular, when contrasting information from independent sources with the information originating from the pharmaceutical industry.

The aim of the present study was to explore GPs'opinions on publicly- and industry-provided drug information. This may contribute to identifying target areas for improving evidence-based drug information to GPs from the DTCs and the pharmaceutical industry.

## Methods

This cross-sectional study was conducted in 2004 using a questionnaire that was answered by 368 GPs at 97 primary-health care centres {PHCC} mainly in the southern part of Sweden, including Stockholm. All 29 Swedish DTCs were invited and eight DTCs, representing 173 PHCCs, accepted participation. We chose to invite through the DTCs as they were in charge of giving public drug information to GPs. Non-participating DTCs were occupied with other projects, had lack of informers and time to participate. The participating DTCs in turn invited primary health care centres and their GPs to participate. Participating PHCCs had 462 GPs in total.

Regional ethical review board approved the study in 2004 (129-04).

### Questionnaire

A questionnaire (Table 1) was developed in cooperation with six experienced colleagues in a network dealing with medication in the Swedish Association of General Practice. Seven questions dealt with origin of drug information, the amount, quality, usefulness and if so, how soon the information proved to be useful. One open-ended question asked for useful examples. Finally GPs were asked to approve or disapprove to statements whether the work of industry and public authorities, respectively, was to i; improve GPs' knowledge of drugs, ii; to influence cost of medication (public authorities) or iii; sales of drugs (industry). All questions (except one) were Likert scales anchored from 1 to 7. Open-ended questions (item 6) were categorized and the responses in each category were counted. The first edition was elaborated together with the above mentioned network of six experienced colleagues. To increase reliability a first edition of the questionnaire was tested on the colleagues and revised. The final version of the questionnaire was sent to each PHCC director for distribution. Non-responders were twice reminded with a two-week interval via the director of the PHCC.

**Table 1:** Topics in questionnaire on attitudes to drug information and responses (mean and median) from 368 GPs

	Item*	N (%)**	Mean (SD)***	Median (IQR)****		
1	Where do you mostly get information about drugs? (pharmaceutical industry --- public authorities)	313 (85.1)	3.7 (2.7)	4.0 (3.0)		
2a	What is your opinion on the amount of drug information you get from public authorities? (too scarce --- too extensive)	313 (85.1)	3.1 (1.2)	3.0 (2.0)		
2b	What is your opinion on the amount of information from the pharmaceutical industry? (too scarce --- too extensive)	314 (85.3)	5.6 (1.3)	6.0 (2.0)		
3a	What is your opinion on the quality of drug information from public authorities? (very poor --- excellent)	313 (85.1)	5.2 (1.4)	6.0 (1.0)		
3b	What is your opinion on the quality of drug information from the pharmaceutical industry? (very poor ---excellent)	313 (85.1)	3.7 (1.4)	4.0 (2.0)		
4a	Do you usually find drug information from public authorities useful? (Not at all --- a great deal)	314 (85.3)	5.4 (1.3)	6.0 (1.0)		
4b	Do you usually find drug information from the pharmaceutical industry useful? (Not at all --- a great deal)	312 (84.8)	3.8 (1.4)	4.0 (2.0)		
5a	If you usually find drug information from public authorities useful - how soon does it prove to be useful? (Later on --- immediately)	306 (83.2)	4.5 (1.4)	5.0 (2.0)		
5b	If you usually find drug information from the pharmaceutical industry useful -how soon does it prove to be useful? (later on --- immediately)	303 (82.3)	4.3 (1.3)	4.0 (2.0)		
6a	If you usually find drug information from public authorities useful - please give some examples.	-----	-----	-----		
6b	If you usually find drug information from the pharmaceutical industry useful- please give some examples.	-----	-----	-----		
To what extent do you agree with the following four statements?		-----	-----	-----		
(do not agree at all --- agree totally)						
7a	The main task of the drug information from public authorities is to increase my knowledge of drugs.	314 (85, 3)	5.7 (1.4)	6.0 (2.0)		
7b	The main task of the drug information from the pharmaceutical industry is to increase my knowledge of drugs.	313 (85, 1)	3.5 (1.7)	3.0 (3.0)		
7c	The main task of the drug information from public authorities is to influence the cost of medication for providers.	313 (85, 1)	5.0 (1.5)	5.0 (2.0)		
7d	The main task of the drug information from the pharmaceutical industry is to influence the company's sales.	313 (85, 1)	6.2 (1.1)	7.0 (1.0)		

*All questions except 6a and 6b were Likert scales anchored from 1 to 7 with 1 representing the alternative seen left in brackets below the item and 7 as the alternative seen right in brackets below the item. Society = Societal information about drugs from e.g. drug and therapeutic committees, The Medical Products Agency, The National Board of Health and Welfare, The Swedish Council on Technology Assessment in Health Care, The Swedish Drug Compendium, postal advertisement, information during lunch, educational events.

**The questions were at most answered by 368 GPs, frequency varying for different questions.

***Mean with standard deviation within parenthesis

****Median with inter quartile range within parenthesis

### Statistical analysis

For each question, we calculated a dichotomized outcome variable, which was 1 if the category of the answer was larger or equal to the median, and 0 if otherwise [23]. Independent variables were the physician's sex, work experience, age and whether the physician worked in the public sector or in private enterprise. These independent variables were treated as fixed effects in a multiple logistic regression model. As the physician's age and GP's experience were strongly correlated they could not be included in the same model. Thus, each question was analysed twice, first by age and all other independent variables except experience, and vice versa.

A multilevel model was used to analyze the correlation in the opinions of GPs working at the same PHCC or in an area connected with the same DTC. We included GPs' workplace, located within areas belonging to a particular DTC as random effects in the multilevel logistic model. For each random effect, the variance between groups (PHCC or regions) was transformed into an intra-class correlation coefficient which could be interpreted as the proportion of variation of the dependent variable that could be explained by variation in the random effect variable. Two-sided p-values were presented both for fixed and random effects. Significance was set at 0.05. Data was entered into the statistical programme Epi-info 3.4.3 (CDC, Atlanta, U.S.A.) which was used for descriptive analysis. For multi-level logistic analysis we used MLwiN v2.00 (Thousand Oaks, Calif: SAGE; 1999).

## Results

Response rates were 28% for the DTCs (8/29), 56% for the PHCCs (97/173) and 80% for the GPs (368/462), totally 218 males and 130 females (20 missing values for sex). Six PHCCs were privately owned and 91 were in the public sector. Mean experience as a GP was 13.9 years (n = 363, SD 8.6). Mean age was 51 years (SD 7.5) for male physicians and 49 years (SD 7.2) for female. There was a mean of 4.8 GPs per PHCC (SD 2.5) with a range of 1-15 GPs. Six PHCCs had only one GP employed.

Most GPs, about 85%, thought that the amount of information from the drug industry was too extensive (item 2b), that the drug information from public authorities was of high quality and useful (item 3a+4a), that public authorities' main task was to increase the physician's knowledge of drugs (item 7a) and that the main purpose of the industry providing drug information was to promote sales (item 7d) (Table 1).

In item 1, 4a, 4b and 7a, a consistent finding was that male GPs were more orientated towards industry-provided drug information, compared to female GPs (Table 2). In item 2b, 4b and 7d a consistent finding was that older GPs and those with longer experience were more positively orientated towards industry-provided drug information compared to younger GPs and those with less work experience (Table 2). Older GPs and those with longer work experience did to a greater extent consider that increasing their knowledge of drugs was a major task for public authorities (item 7a, Table 2). Furthermore, GPs with greater work experience considered to a lesser extent that the aim of public authority information was to influence the cost of medication to society (item 7c, Table 2). In item 3b there was a weak tendency and in item 4b a more consistent finding that private sector GPs ranked the quality and usefulness of industry-provided drug information higher as compared to GPs in the public sector (Table 2). In item 7d GPs in the public sector to a larger extent considered the main task of the industry to increase their sales compared to GPs in the private sector (Table 2).

**Table 2:** Questionnaire responses related to sex, age, work experience, sector and geographical area

		Fixed effects				Random effects	
**Item^a^**	**N^b^**	**Sex (female/male)^c^**	**Age^cf^**	**Work experience^cf^**	**Sector (Public/Private)^c^**	**PHCC^d^**	**Area^e^**

1	292	1.92 (1.17-3.14)**	1, 01 (0.98-1.04)	-----	1.38 (0.57-3.38)	0.039	0
	291	1.97 (1.19-3.26)**	-----	1.02 (0.99-1.06)	1.27 (0.51-3.20)	0.052	0
2a	292	1.64 (0.97-2.79)	1.00 (0.96-1.03)	----	1.06(0.39-2.89)	0.075	0.022
	291	1.66 (0.97-2.83)	-----	1.00 (0.97-1.03)	1.07 (0.40-2.87)	0.065	0.026
2b	292	1.35 (0.80-2.26)	0.96 (0.92-0.99)**	----	1.45 (0.54-3.89)	0.082	0.01
	291	1.37 (0.81-2.29)	-----	0.98 (0.95-1.00)*	1.50 (0.56-4.00)	0.087	0.001
3a	291	1.46 (0.88-2.43)	0.99 (0.97-1.00)	----	1.67 (0.63-4.43)	0.054	0.031
	290	1.49 (0.90-2.48)	-----	0.99 (0.96-1.02)	1.55 (0.58-4.18)	0.060	0.033
3b	291	1.29 (0.78-2.13)	1.03 (1.00-1.07)	----	0.37 (0.13-1.05)	0.092	0
	290	1.31 (0.79-2.17)	-----	1.02 (0.99-1.05)	0.36 (0.12-1.04)	0.094	0
4a	292	2.06 (1.24-3.40)**	0.99 (0.97-1.02)	----	1.19 (0.50-2.82)	0.022	0
	291	2.07 (1.24-3.44)**	-----	1.00 (0.97-1.03)	1.15 (0.48-2.77)	0.029	0
4b	291	0.55 (0.32-0.94)*	1.04 (1.01-1.08)*	----	0.20 (0.05-0.76)*	0.163*	0.036
	290	0.57 (0.33-0.98)*	-----	1.04 (1.00-1.07)**	0.19 (0.05-0.74)*	0.169*	0.034
5a	286	0.82 (0.51-1.33)	1.02 (0.99-1.05)	----	0.95 (0.42-2.16)	0	0
	285	0.55 (0.26-1.14)	-----	1.00 (0.95-1.04)	1.00 (0.40-2.49)	0	0
5b	283	1.04 (0.61-1.80)	1.01 (0.98-1.05)	----	0.78 (0.30-2.04)	0	0
	282	1.00 (0.58-1.74)	-----	1.00 (0.97-1.03)	0.79 (0.30-2.08)	0	0
7a	292	1.76 (1.02-3.03)*	1.04 (1.00-1.08)*	----	1.27 (0.49-3.24)	0.057	0
	291	1.78 (1.03-3.07)*	-----	1.04 (1.01-1.07)**	1.15 (0.46-2.91)	0.047	0
7b	291	1.17 (0.70-1.94)	1.01 (0.98-1.05)	----	0.59 (0.22-1.61)	0.062	0
	290	1.20 (0.72-2.01)	-----	1.01 (0.98-1.04)	0.58 (0.21-1.59)	0.065	0
7c	291	1.17 (0.68-2.03)	0.97 (0.93-1.01)	----	0.97 (0.33-2.89)	0.101	0.016
	290	1.12 (0.64-1.97)	-----	0.97 (0.94-1.00)*	1.02 (0.33-3.11)	0.112	0.020
7d	291	1.10 (0.68-1.78)	0.96 (0.93-0.99)**	-----	2.70 (1.12-6.49)*	0	0
	290	1.04 (0.64-1.69)	-----	0.97 (0.94-1.00)*	2.80 (1.16-6.75)*	0	0

*p < 0.05,**p < 0.01

^a ^Question according to Table 1

^b ^Number of responders with response for all items included in the regression model

^c ^Odds ratio (95% confidence interval) --- two sided p-value.

First line when age is included as independent variable and second line when working time is included as independent variable.

^d ^Variation among primary health care centres is transformed to intra-class correlation. ICC --- One sided p-value for variation

^e ^Variation among regions is transformed to intra-class correlation. ICC --- One side p-value for variation

^f ^Odds ratio for an increase of one year in age or working time

For most items, the random effects describing correlations within PHCCs or regions adhering to the same DTC were of little importance compared to fixed effects such as sex, age, work experience and sector (Table 2). For all questions, we found that the variation in opinions between different PHCCs was larger than between regions connected to different DTCs, or, in other words, we observed a relatively high correlation of GPs opinions within the same PHCC, regardless of where they worked. This was most prominent how the doctors regarded the usefulness of received information from the pharmaceutical industry (item 4b). The GPs were also asked to describe in their own words different aspects of drug information from public authorities and from the pharmaceutical industry (item 6a and 6b). The former was regarded as useful for the GPs in making scientific judgements on drugs, concerning economic aspects of drug therapy and in providing objective information. The latter was regarded as useful in providing information on new drugs, in making scientific judgements on drugs and providing useful information on how the patient can use the drugs. To provide information of side effects was mentioned as examples of drug information only by a few GPs for both public authorities and the pharmaceutical industry (Table 3).

**Table 3:** Perceived aspects of drug information according to open-ended questionnaire responses (Item 6a and b)

Perceived aspects of drug information	Public authorities (item 6a^a^)	Pharmaceutical industry (Item 6b^a^)
	n = 205^b^	n = 186^b^
Provide drug information and education^c^	5.9% (12)	24% (44)
Provide useful information on how patient can use the drug^c^	3.4% (7)	17% (31)
Provide information of side effects^c^	6.8% (14)	3.8% (7)
Provide economic aspects of drug therapy^c^	16% (33)	1.1% (2)
Provide information on new drugs^c^	3.9% (8)	38% (71)
Provide scientific judgement on drugs^c^	53% (109)	33% (61)
Provide information from public authorities^c^	60% (122)	1.6% (3^d^)
Provided information is objective^c^	12% (25)	0.54% (1)

^a ^Question according to Table 1

^b ^Number of responders to the item

^c ^Responders wrote freely. The response was then classified into categories and counted. One responder can contribute to more than one alternative

^d ^GPs sometimes perceive that pharmaceutical industry present information from public authorities essential for their product.

## Discussion

### Main findings

GPs in the study considered that drug promotion from the industry was too extensive and that drug information from public authorities was useful and of good quality. They also stated that the main task of public authorities was to promote the GPs knowledge of drugs and that the industry's main task was to promote sales. Male, older, and GPs with longer work experience or working in the private sector were more positive towards industry-provided information whereas female GPs to a greater extent valued information from public authorities.

### Strengths and limitations of the study

Sample size or power was not calculated in the study. For some items the number of answering GPs might be too small.

Eight out of 29 DTCs in Sweden took part in this study. The limited number of participating DTCs and the fact that the northern part of Sweden was represented only by one geographical area in the study can be regarded as a limitation. On the other hand 4.2 out of 9.1 million inhabitants in Sweden lived in the geographical regions surrounding the participating DTCs. Large and medium-sized cities as well as rural areas were represented. The proportions of our study group (37% female and 63% male) were similar to all GPs in Sweden 2004 and 2005 (38% and 62%**)**[24]. Despite of the consistency of figures between the groups we cannot ignore the possibility of reduced internal validity due to selection bias. Further, bias due to interest and possibilities to take part in education e.g. concerning EBM could also influence the results. However, the high number of participating GPs as well as an acceptable response rate could justify the assumption that the results are representative for Swedish GPs. The proportions of male and female GPs in this paper correspond to the whole of Sweden and support the assumption that our sample gives a fairly good representation of Swedish GPs.

### Comparison with existing literature

Literature on the opinions of GPs' relations to the pharmaceutical industry has been published [9,25,26], but scientific knowledge comparing drug information from, respectively, public authorities and industry has been sparse. GPs were accustomed to receiving most of their information from the pharmaceutical industry. Thus, our results showing that the greatest part of the GPs drug information emanates from the industry are not surprising [26,27]. The response that promotion from the industry was considered too extensive was also consistent with the literature [16,18-20]. There are also reasons to believe that many financial ties between industry and medicine are hidden [7].

During the last decades critical voices raised against GPs and other physicians' dependence on the pharmaceutical industry has been augmented. The awareness of physicians on the effects of the relations has been low [9,25]. There is evidence that the relations may have effects on the prescribing, costs and quality [28]. Medical students in Norway seem to be critical and curious to the industry and were influenced by their teachers [29] and today's students might be important to better align physician attitudes with current policy trends in future [9]. There are differences between the UK and Greece/Cyprus on how physicians uptake new medicines with regard to policy trends and costs [15,26]. In the UK GPs have been influenced by government policies by incentives but there is a risk that unintended consequences may ensue due to the cost reduction [15]. In Greece/Cyprus there are no financial incentives to motivate physicians to prescribe generics and these are not prescribed by Greek physicians [26].

GPs have a wide domain of practice in combination with lack of time [30]. This might imply that GPs could be more vulnerable to exposure to pharmaceutical advertisements than other physicians. The amount of new information has been extensive and keeping up to date with best evidence in health is challenging [31]. To be able to maintain a critical appraisal of new medical concepts, e.g. as prehypertension was recognized as diagnose [32], it is important to allow time for continuing education, reading and reflection based on reliable sources [26,27,33]. An informer giving evidence-based knowledge adjusted to the week-day of GPs could perhaps be one appropriate means for this. An American survey showed that significant attitude shifts were seen among physicians concerning perceptions about industry after information on their working methods [33].

A consistent and new finding was that male GPs were more orientated towards industry-provided drug information compared to female GPs. This finding needs further investigation but earlier findings concerning physician gender differences showing that female physicians were more involved and had longer visits [34] as well as differences in prescription patterns [35] indicate the existence of gender differences in this field.

Concerning the opinions on both public authority and industry-supplied drug information services, the role of the information provider, as an important link between the public authorities or the industry and the GP, must be put forward even if the literature is sparse [22]. Public-authority providers in Sweden are often GPs or pharmacists and usually well known and accepted by the GPs, traditionally not putting focus on GPs as customers.

An Australian study on hypertension advertisements implied that the industry lacked elements important for cost-effective care consistent with evidence-based guidelines [36]. In another study exposure to pharmaceutical information was associated with either no effect or adverse effect on prescribing [28]. This implies that physicians could be seen as customers representing medical care in relation to the industry.

Changing medical culture and physician education is an important field to be improved as many physicians still hold positive attitudes toward marketing-oriented activities [9]. The positive attitudes in this study could perhaps partly be explained by some GPs' appreciation of the usefulness of the information provided by the industry on how the patient could use drugs. This was mentioned only by a few GPs as examples of useful information from the public authorities.

From the mid 90 s, the relations between the public health care and GPs on the one hand and the industry on the other became chillier in Sweden. There has long been a largely ignored ethical agreement between health care and industry concerning education, information and clinical trials. A debate concerning regions' task of taking economic responsibility for drugs, instead of the state, was accelerating. In due course this led to a new and more efficient agreement which was initiated in 2004 [37]. Our finding that older GPs and those with more years of experience were more positive towards industry-provided promotion might be explained by their experience from a closer relationship that earlier was accepted. These contacts are still accepted in other countries [9,26] and perhaps attitudes of younger GPs in Sweden are more like those in the UK [15]. That older and more experienced GPs also considered that increasing their knowledge was a major task for public authorities could perhaps be explained by their knowledge that the industry is prone to sell their products but not to give a wider spectrum of information. An Australian print advertisement study found that advertisements provide some but not all key messages for a guide-line concordant care [36]. GPs working in private enterprise were more positive towards the industry in this study. This might be a reflection of entrepreneurs having a more sceptical approach to public authorities.

There were greater differences in use of industry-provided information between PHCCs, than between geographical areas. Since the new agreement between regions and the pharmaceutical industry was settled [37], the tradition of seeing pharmaceutical representatives at lunchtime has been reduced at most but not at all PHCCs. This might explain the high ICC for PHCC in item 4b (Table II). Leadership forming policies at the centres could also have other impacts on attitudes [38] as could work conditions and culture among GPs and other staff.

## Conclusions

As Swedish GPs considered drug promotion from the industry to be too extensive some kind of incentives could be considered for PHCCs that actively reduce drug promotion from the industry.

Female GPs valued information from public authorities to a much greater extent than male GPs and this difference should be taken into consideration when designing evidence-based drug information from public authorities to make implementation easier.

## List of abbreviations

CME: Continuing Education; DTC: Drug and therapeutic committee; EBM: Evidence based medicine; GP: General practitioner; ICC: Intra-class correlation coefficient; PHCC: Primary health care centres.

## Competing interests

Ingmarie Skoglund is former chairwoman of the drug and therapeutic committee in Södra Älvsborg.

The other authors declare that they have no competing interests.

## Authors' contributions

IS conceived the study, acquired the funding, participated in the design of the study, performed the recruitment, enrolment and data collection, participated in the statistical analysis and drafted the manuscript. CB participated in the design of the study and drafting the manuscript. KM performed the statistical analysis and participated in drafting the manuscript. RG participated in the design of the study, statistical analysis and in drafting the manuscript. MM participated in the design of the study, statistical analysis and in drafting the manuscript. All authors read and approved the final manuscript.

## Authors' information

IS: General Practitioner

CB: MD, PhD, professor, GP

KM: PhD, M.Sc. in mathematical statistics

RG: MD, professor,

GP MM: R.PT., professor

## Pre-publication history

The pre-publication history for this paper can be accessed here:

http://www.biomedcentral.com/1472-6963/11/204/prepub
